# Floral Characteristics and Reproductive Biology in Brazilian Melon Accessions: Insights from Commercial and Exotic Varieties

**DOI:** 10.3390/plants14193047

**Published:** 2025-10-01

**Authors:** Nadia Carolina Sanabria-Verón, Cláusio Antônio Ferreira de Melo, Glauber Henrique de Sousa Nunes, Delmira Da Costa Silva, Margarete Magalhães de Souza, Ronan Xavier Corrêa

**Affiliations:** 1 Programa de Pós-Graduação em Produção Vegetal (PPGPV), Universidade Estadual de Santa Cruz, Campus Soane Nazaré de Andrade, Rodovia Jorge Amado, km 16, Ilhéus CEP 45662-900, BA, Brazil; 2 Departamento de Ciências Agrárias e Ambientais, Universidade Estadual de Santa Cruz, Campus Soane Nazaré de Andrade, Rodovia Jorge Amado, km 16, Ilhéus CEP 45662-900, BA, Brazil; clausiomelo@gmail.com; 3 Departamento de Ciências Vegetais, Universidade Federal do Semi-Árido (UFERSA), Av. Francisco Mota, 572 Bairro Costa e Silva, Mossoró CEP 59625-900, RN, Brazil; glauber@ufersa.edu.br; 4 Departamento de Ciências Biológicas, Universidade Estadual de Santa Cruz, Campus Soane Nazaré de Andrade, Rodovia Jorge Amado, km 16, Ilhéus CEP 45662-900, BA, Brazil; delmira@uesc.br (D.D.C.S.); souzamagg@yahoo.com.br (M.M.d.S.); ronanxc@uesc.br (R.X.C.)

**Keywords:** melon, flower variability, pollen viability, pollination, stigma receptivity, pistil morphology

## Abstract

Melon has great economic importance in Brazil, and flower development is the basis for fruit and seed production. The objective of this study was to elucidate the variability of flowering characteristics and to compare qualitative and quantitative reproductive variations in relation to pollen viability and stigmatic receptivity in 21 genotypes, which includes 15 Brazilian accessions. In addition, we evaluated the influence of time on the growth of the pollen tube and its arrival at the ovule in vivo at different intervals (1 h, 2 h, 3 h) after hand pollination in three commercial varieties, one exotic accession, and two intervarietal hybrids, by epifluorescence technique. Three groups were distributed by the clustering method of Scott–Knott at 5% probability; group III included only commercial varieties for the flower width descriptor. *C. melo* germplasm presented 81% andromonoecious plants and 19% trimonoecious plants. Through the multivariate strategy, these 21 genotypes were distributed into six groups with distinct reproductive characteristics, and male flowering was accelerated compared to female flowering. Regarding pollen viability, it was greater than 95% according to staining methods. Pollen germination rate in vivo was affected by time, with an almost 12.5% increase between 1 h and 3 h after hand pollination, and the in vivo pollen germination in hybrids was lower than in commercial varieties. Brazilian accessions, despite stability in pollen viability and stigma receptivity, have great differences in reproductive terms, such as variations in the quantitative and qualitative characteristics of floral pieces and flowering. This work contributes to the knowledge on varieties, hybrids, exotic accession, and Brazilian melon germplasm by characterizing some of their main agricultural traits, such as reproduction floral biology, and opens up prospects for yield evaluation in plant breeding programs.

## 1. Introduction

*Cucumis melo* L., a member of the Cucurbitaceae family, represents one of the most economically important species within a group that encompasses approximately 800 species. Melon stands out for its remarkable levels of polymorphism and genetic diversity, which are predominantly expressed in fruit, flower, and leaf phenotypes [1,2,3]. By 2023, Brazil ranked as the fifth-largest melon producer globally and the first in the Americas, with a production volume of 862.387 tons [4]. Melon cultivation in Brazil is concentrated in the northeastern region, particularly in the states of Ceará and Rio Grande do Norte [5]. The cultivated area expanded from 18.870 hectares in 2010 to 30,635 hectares in 2023, consolidating this region as the country’s leading melon-producing area [5]. The most widely cultivated sweet melon types in Brazil belong to the botanical groups Cantalupensis, Reticulatus, Inodorus, and Makuwa. Among the commercial varieties, Yellow, Piel de Sapo (PS), Honey Dew, Cantaloupe, Galia, and Charentais dominate the Brazilian market [6,7].

In cultivated commercial melons, andromonoecy is the predominant reproductive system, occurring in approximately two-thirds of traditional cultivars or landraces, as well as in monoecious collections [7]. Sexual expression in melon is genetically controlled by the interaction of two major genes, a and g, whose allelic combinations result in four distinct genotypes: hermaphrodite (aagg), gynoecious (AAgg), monoecious (A_G_), and andromonoecious (aaG) [8]. In andromonoecious melon plants, male flowers are produced along the main stem and lateral branches, whereas bisexual flowers develop exclusively in the leaf axils of lateral branches. Importantly, only bisexual flowers give rise to fruit development, highlighting their critical role in melon reproductive success [9].

The first step in classical breeding is knowing aspects of reproductive biology. Currently, little is known of the melon reproductive biology, yet this knowledge is essential for pollinations and for increasing the chance of successful fruit formation, which depends on effective pollination, where pollinators play a crucial role in depositing pollen grains onto the stigma surface [10]. The pollen–stigma interaction represents the initial step of the reproductive process, triggering pollen tube growth toward the ovary. During this process, pollen tubes compete for space and resources within the transmitting tissue, and only those originating from genetically compatible pollen grains succeed in reaching the ovary and fertilizing the ovules [11,12]. The remarkable precision with which pollen tubes navigate through the style reflects the complexity of the guidance mechanisms involved, as extensively described in *Arabidopsis thaliana* [13].

Pollen viability and germination assays are widely employed in cytogenetic, physiological, biochemical, molecular, and proteomic studies across diverse plant species, serving as essential tools for advancing knowledge in reproductive biology and plant breeding [14]. In reproductive studies, fluorescence microscopy has become a reliable technique for monitoring pollen tube growth and analyzing floral anatomy [15,16]. Similar to many plant species, melon pollen tubes synthesize callose, a polysaccharide that readily absorbs aniline blue and emits fluorescence when exposed to blue or ultraviolet light, facilitating detailed visualization of pollen tube pathways [17,18,19].

The knowledge of the reproductive relationships between accessions can help select the optimal genetic improvement strategies, especially in designing crosses among contrasting varieties for multiple traits [20], and the study of pollination biology is a tool that can save time and effort in plant breeding programs [21,22].

Although flowers are theoretically considered morphologically constant within a species, quantitative variations frequently occur among closely related species and may also be observed within the same genotype, among individuals growing under similar environmental conditions, or across genotypes from different populations [23]. The cucurbit germplasm collection maintained by the Federal University of the Semi-Arid Region (UFERSA, Brazil) comprises a representative sample of melon accessions cultivated by smallholder farmers, part of which has been previously characterized in reproductive biology studies. In addition, seven Brazilian accessions were found to have similarities with PI161375 for resistance to the cucumber mosaic virus (CMV) [24]. These findings are essential, considering that the CMV constitutes a potential threat to many crops, especially cucurbits, and therefore to melon cultivation, and that the search for resistance genes should be carried out by evaluating accessions preserved in germplasm banks (GBB). Researchers in the field of genetic improvement should be involved in everything from the search for sources of resistance to the development of resistant cultivars [25]. This study seeks to expand that knowledge by integrating floral and reproductive descriptors to deepen the understanding of morphological variability within the collection. Such an approach provides valuable insights into the genetic diversity preserved in the germplasm bank, supporting its application in breeding programs and facilitating the selection of parental lines for future crosses. Therefore, the objective of this research was to assess the variability in floral characteristics and reproductive behavior across different melon accessions, focusing on qualitative and quantitative variations in pollen viability, stigmatic receptivity, and floral morphological traits. Additionally, the study evaluated the influence of time after manual pollination on pollen tube emission and growth in a diverse set of genotypes, including commercial varieties, Brazilian accessions, exotic materials, and intervarietal hybrids, through detailed anatomical analysis and epifluorescence techniques.

## 2. Material and Methods

### 2.1. Plant Materials

Melon plants used in this study were obtained from the Cucurbitaceae Germplasm Bank of the Federal University of Semiarid (UFERSA), Rio Grande do Norte, Brazil, consisting of 15 traditional Brazilian accessions, 3 commercial varieties, 1 exotic accession and 2 intervarietal hybrids (21 genotypes). The experiment was conducted in a greenhouse at UESC, Ilhéus, Brazil (14.7980° S, 39.1764° W); Dec/2019–Mar/2022; SisGen registration A1FF824. The seeds were placed to germinate in a polystyrene cup filled with commercial substrate and soil in a 1:1 ratio. Transplantation was performed 15 days after sowing into a 10 L polystyrene pot, and fertilization management was performed via chemical fertilization and irrigation according to [26]. All experimental assessments were carried out in a completely randomized model, and six flowers of each accession were evaluated.

The main botanical groups are classified by their characteristics of leaf color, type of flower, and characteristics of their fruit such as sweetness, aroma, and if they are climacteric. For genetic analyses, the germplasm was distributed into six genetically distinct groups: *conomon* (A9, A11) and *momordica* (A30), which were described as not sweet. The groups classified as sweet were *cantalupensis* (A1, A6, A7, A18, A24), *reticulatus* (Galia), *inodorus* (A43, A44, Piel de Sapo, Ouro), and *chinensis* PI161375 (Sonwang Charmi—SC). The following accessions were not classified: A19, A27, A35, A50, D2, PI161375 x Galia, and PI161375 x Ouro [7,27,28].

### 2.2. Floral Descriptors

Floral descriptors were determined for statistical measures and comparisons among accessions. Six quantitative variables were measured to the nearest 0.01 mm with a digital caliper. The flower reproductive traits were analyzed in fresh flowers harvested from the field. From each randomly chosen plant, one flower was dissected under a binocular stereoscopic microscope to separate the corolla, receptacle, androecium, and gynoecium and these parts were then measured. The species has yellow, bell-shaped flowers, and the floral descriptors were as follows: *petal length* (PL), distance between the two longitudinal margin ends; *flower width* (FW), largest horizontal distance between the open flower edges; *receptacle length* (RL), distance between the two longitudinal ends; *bract length* (BL), from the insertion of bracts into the receptacle base; *receptacle width* (RW), largest dimension of the receptacle diameter; and *flower length* (FL), corresponding distance between the two longitudinal ends.

In pistillate flowers, the following descriptors were measured: *ovary length* (OL), corresponding to the length between the base and stylus ends; *ovary width* (OW), corresponding to the largest horizontal length; *stigma length* (SL), from the insertion of the ovary stigma to the stigma end; and *stigma width* (SW), the horizontal length between stigma borders.

### 2.3. Phenology

The descriptor *Number of male flowering days* was considered as the number of days to the anthesis of the first staminate flower in 50% of the plants in the plot. Plants were categorized as follows: precocious (<29 days after sowing), medium (30 to 34 days after sowing), and late (>35 days after sowing). The descriptor *Number of female flowering days* after sowing was considered as the number of days to the anthesis of the first pistillate flower in 50% of the plants in the plot. The number of days for flowering was classified as follows: precocious (<40 days after sowing), medium (41 to 45 days after sowing), and late (>46 days after sowing).

### 2.4. Pollen Viability

For pollen analysis, mature flowers at anthesis were randomly collected at 7:00 am, and pollen grains (PG) were placed in Petri dishes, duly protected with aluminum foil, closed, and taken to the laboratory of the (the institution name will be informed after peer review of this manuscript). Viability was assessed using the staining method with carmine acetic acid. Pollen grains were gently placed over a drop of dye at room temperature (RT) and then observed under a light microscope for morphological appearance and colorability. Stained PG with intact cytoplasm were considered viable. In each drop of suspension, four fields of view under the microscope were analyzed, which were equivalent to four repetitions. Images were captured with a digital camera Olympus C-7070, 7.1 MP, coupled to a light microscope Olympus CX41 (Tokyo, Japan). 

### 2.5. Stigma Receptivity and Pistil Morphological Description

For stigma receptiveness, the mature staminate flower at anthesis was dissected and subsequently submerged in 1% benzidine solution in 60% ethanol and 3% hydrogen peroxide [29] at RT. The stigma was considered receptive when the color changed due to oxidation and bubbles formed upon contact with the solution [30]. At least four flowers were evaluated per accession. For pistil morphological description observations, bisexual flowers were fixed in FAA 50 (5% formalin, 5% acetic acid, 90% of ethanol 50%) [31]. After three washes with water, the pistils were cut into cross sections with a scalpel, mounted on slides, and measured in mm with a digital caliper. Images were captured with a digital camera Olympus C-7070, 7.1 MP, coupled to a light microscope Olympus CX41 (Tokyo, Japan).

### 2.6. Pollen Tube Growth and Arrival in Ovules In Vivo

The number of pollen tubes growing in the melon stigma in vivo and the arrival of pollen tubes in ovules were observed according to [32], with some adaptations. A completely randomized 6 × 3 factorial design was used, with six accessions (PI161375, Piel de Sapo, Ouro, PI161375 x Ouro, Galia, PI161375 x Galia) and three different intervals after hand pollination. From each accession, five male flowers were emasculated, and PG were deposited on the stigmas of pistillate flowers. After hand pollination of the flowers, they were fixed in FAA 50 at 1 h, 2 h, and 3 h, then, clarified and softened in a strong 5N sodium hydroxide solution for 24 h at RT. Staining was performed in a 0.1% solution of aqueous soluble aniline blue dye dissolved in 0.1N K_2_HPO_4_ for 10 min. Longitudinal dissections of pistils were performed and mounted on slides. Observations were made with UV microscope Olympus CX41 with Olympus C-7070, 7.1 MP (Tokyo, Japan) camera (wavelength of about 360 nm) in a dark room. PG that germinated and produced a viable pollen tube, whose length was greater than the pollen diameter, was considered viable [33]. The pollen–pistil relationship was considered compatible when pollen tube growth reached the ovule. At least five bisexual flowers were analyzed from each genotype, totaling 30 flowers.

### 2.7. Internal Structures of Bisexual Flowers

For the morpho-anatomical observations, hermaphrodite flowers were fixed in FAA 50 (formaldehyde, acetic acid, and ethanol solution 50%), then dehydrated using ethanol series, embedded in glycol methacrylate at 50 °C for 24 h (Historesin Leica, Nussloch, Germany), and sectioned at 5 µm thickness using a rotary microtome (RM 2145, Leica, Germany) with glass knife. The sections were stained with Toluidine Blue O (C.I. 52040) 0.05 % in 0.1 M phosphate buffer solution at pH 4.7 [34].

### 2.8. Statistical Analyses

The values expressing the number of viable pollen grains were transformed using arcsin (x √100) prior to statistical analysis. The means were tested by the Scott–Knott clustering method at a 5% probability. The error was determined by the statistical program, InfoStat/Professional, version 2013 [35]. For quantitative floral descriptors, an analysis of variance (ANOVA) and a Ward–MLM multivariate analysis was performed. The average values of the quantitative variables were included in the analysis together with the qualitative flowering data and flower types. The Ward–MLM multivariate procedure was performed using the distance matrix obtained by the logarithmic function of the Gower coefficient [36] on SAS (2000) *software* analysis platform.

## 3. Results

### 3.1. Floral Characteristics

**Figure 1:**
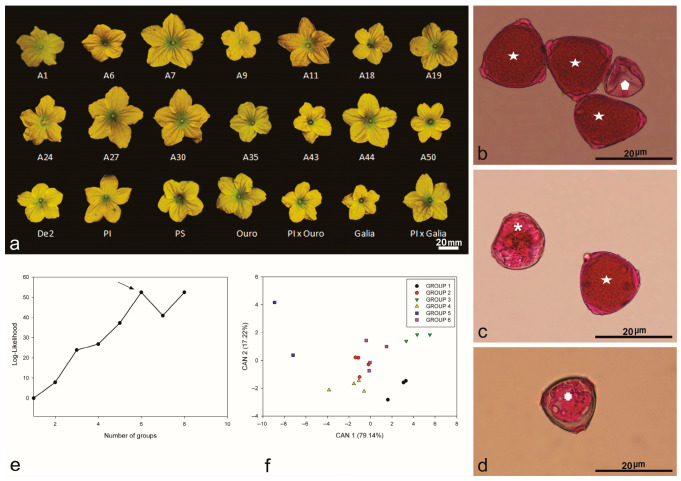
Flower of Brazilian melon accessions: (**a**) morphology of staminate flowers from commercial (A1, A6, A7, A9, A11, A18, A19, A24, A27, A30, A35, A43, A44, A50, De2) varieties (Galia, Ouro, Piel de Sapo), hybrids (PI161375 x Galia, PI161375 x Ouro), and exotic (PI161375) accessions. Pollen grain viability stained with carmine acetic acid dye: (**b**) full (star) and empty (polygon) pollen grains, not viable for pollination; (**c**) collapsed pollen grain considered as sterile (asterisk) and viable pollen grain (star); (**d**) sterile unviable pollen grain (cross). Multivariate analysis by the Ward–MLM strategy: (**e**) logarithmic function probability (log-likelihood) showing the formation of six groups of melon germplasm, indicating greater amplitude (arrow); (**f**) first two canonical variables (CAN) in the melon germplasm analyzed, forming six statistical groups.

The diversity in floral characteristics was demonstrated among 21 melon genotypes (Figure 1). All genotypes presented staminate flowers, although considerable variability was observed in their phenotypic descriptors (Figure 1a, Table 1). Notably, flower size, a key trait for pollinator attraction, varied significantly among the genotypes.

Significant differences (*p* < 0.05) were observed among floral descriptors, especially regarding staminate flower size (Table 1). Petal length ranged from 11.81 mm in the Galia accession to 18.07 mm in accession A27, with an average of 11.81 mm and a coefficient of variation of 12.80% (Table 1). Flower width was greatest in accession A7 (35.01 mm) and smallest in Piel de Sapo (23.46 mm), with an overall mean of 29.44 mm. This variation in flower width highlights the quantitative floral traits observed among accessions (Figure 1a).

The Scott–Knott test separated the melon accessions into three distinct groups. Group I included seven Brazilian accessions; group II comprised ten accessions, encompassing hybrids and their exotic progenitor; and group III contained only commercial varieties (Piel de Sapo, Galia, and Ouro).

Receptacle length ranged from 4.61 mm in accession A43 to 7.54 mm in accession A7, averaging 5.86 mm. Bract length was greatest in accession A7 (5.36 mm) and smallest in accession A35 (2.67 mm), with an overall mean of 3.91 mm. Receptacle width varied from 2.75 mm (accession A35) to 3.79 mm (accession A44). Additionally, flower length measurements were highest in accession A7 (25.11 mm) and lowest in accession A43 (17.33 mm) (Figure 1, Table 1).

A combined Ward–MLM approach was used to assess the genetic diversity in the studied germplasm, drawing on floral descriptors, flowering time, and flower types. The germplasm was subsequently distributed into six genetically distinct groups, consistent with [7]. The optimal group count was determined by the largest increase in the likelihood function, which occurred at six groups, with an increment of 52.502 (Figure 1e). The shortest genetic distance was observed between groups 2 and 6, whereas groups 1 and 5 were the most distant (Figure 1f).

When variation was plotted using the first two canonical variables, clear separations emerged among the groups. These two variables together explained 96.36% of the overall variation in floral and reproductive traits (Figure 1f).

Group 1 comprised three accessions, whereas group 6 contained four. Ouro and PI161375 x Ouro were assigned to group 4 alongside A19 and A50. Although multiple floral descriptors influenced the variation observed via the Ward–MLM analysis, flower width emerged as the primary trait explaining germplasm differentiation, whereas flower length contributed the least. Genetic group 3, represented by A1, A7, A24, and A27, exhibited the most uniform flowering time and flower type.

Analysis of qualitative variables related to flowering time and flower types showed that group 1 was largely homogeneous, except for accession A35. In groups 1, 2, and 3, male flowering time ranged from precocious to medium, while groups 4 and 5 were characterized by late male flowering only. Pistillate flowering spanned from precocious to medium in groups 1, 2, 3, and 4, and from precocious to late in group 6. In contrast, group 5, which included only commercial cultivars (Piel de Sapo and Galia), consistently exhibited late pistillate flowering. Groups 4 and 6 were the most heterogeneous (Table 1).

**Figure 2:**
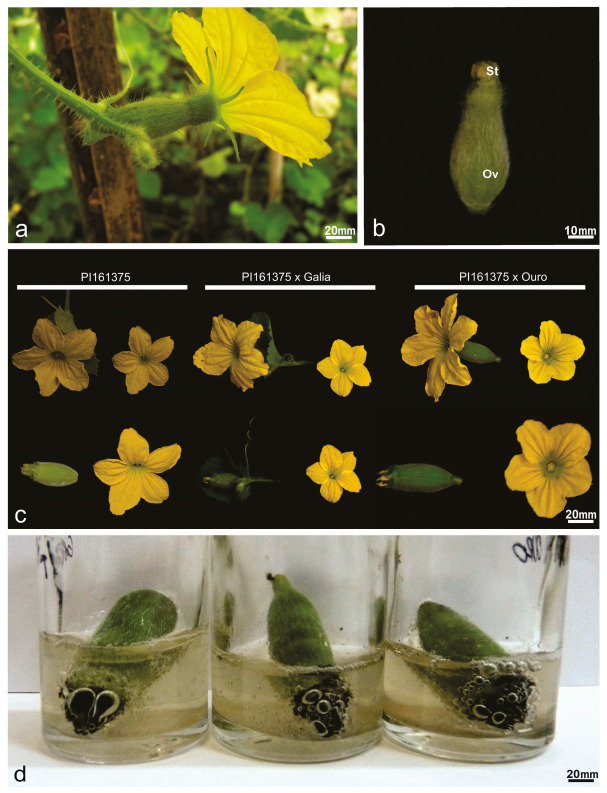
Flowers of *Cucumis melo* germplasm accessions. (**a**) Mature hermaphrodite melon flower after emasculation. (**b**) Gynoecium mature young hermaphrodite flower in frontal view showing the stigma (St) and the ovary (Ov). (**c**) Comparison of hermaphrodite perfect hybrid flowers and the parental PI161375. (**d**) Stigma of hybrids bubbling in benzidine solution and 3% hydrogen peroxide; the black color at the stigma end is the result of an oxidation process and indicates receptivity.

Three types of flower reproductive structures (pistillate, staminate, and bisexual) were identified in the germplasm collection. However, plants in groups 2, 3, and 5 produced only staminate and bisexual flowers, whereas those in groups 1 (A35), 4 (A19, A50), and 6 (De2) exhibited all three types (Table 1, Figure 1). Bisexual flower descriptors varied significantly among three commercial varieties, one exotic accession, and two intervarietal hybrids (Table 2, Figure 2a,b).

Within this set of genotypes, the hybrid PI161375 x Galia presented the greatest ovary length (24.65 mm), followed by Piel de Sapo (21.07 mm), which differed statistically from PI161375 (16.68 mm) and PI161375 x Ouro (17.24 mm). The smallest ovary lengths were recorded in Galia (13.18 mm) and Ouro (15.13 mm). Ovary width was largest in Piel de Sapo (11.05 mm) and smallest in Ouro (7.15 mm). Stigma length ranged from 4.37 mm in PI161375 x Ouro to 5.13 mm in PI161375, while stigma width varied from 3.51 mm in Galia to 5.12 mm in PI161375 (Table 2).

### 3.2. Pollen Viability

The pollen viability assay revealed no statistically significant differences among the melon accessions tested (*p* < 0.05; Table 1). Examination of pollen grains (PG) from staminate flowers across all genotypes identified three distinct morphological categories: (i) intact pollen grains, indicative of viability (Figure 1b, short arrow); (ii) empty pollen grains, interpreted as non-viable (Figure 1c, short arrow); and (iii) collapsed pollen grains, also non-viable (Figure 1d).

### 3.3. Stigma Receptivity and Pistil Morphological Description

To assess stigmatic receptivity, mature bisexual flowers were examined (Figure 2a), with petals removed to fully expose the stigma surface (Figure 2b, arrowhead). Comparative analyses between hermaphroditic flowers of the parental line PI161375 and their hybrids revealed distinctive morphological traits specific to pistillate structures (Table 2, Figure 2c). All evaluated accessions, including hybrids, demonstrated clear stigmatic receptivity (Figure 2d).

**Figure 3:**
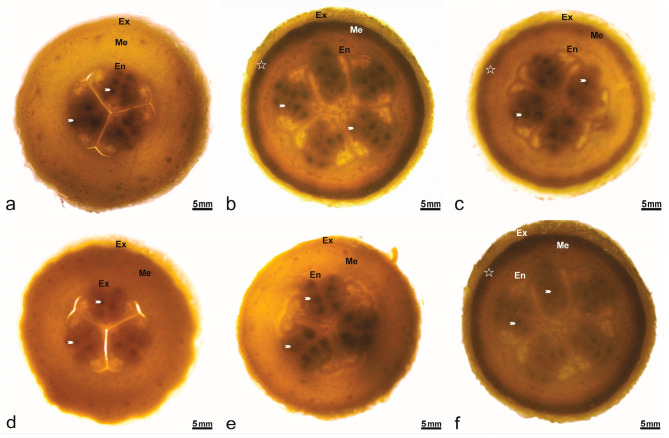
Transversal section of the ovary in *Cucumis melo* accessions. Numerous ovules (arrowheads) per loculus in the ovary covered by epidermis (Ep), contain fleshy mesophyll parenchymatous (Me) with layers of supporting tissues (star). (**a**) Ouro. (**b**) PI161375. (**c**) PI161375 x Ouro. (**d**) Piel de Sapo. (**e**) Galia. (**f**) PI161375 x Galia.

In *Cucumis melo*, the pistillate flower is characterized by a multicarpellate gynoecium, responsible for seed formation within the lower ovarian cortex (Figure 3). Detailed anatomical observation revealed plurilocular ovaries containing developed ovules (Figure 3a, white arrow). The three commercial cultivars (Figure 3a,d,e) consistently exhibited three ovarian locules, whereas PI161375 (Figure 3b) and its hybrid progeny presented five. Furthermore, the exocarp layer was notably more developed in hybrids derived from PI161375 (Figure 3c,f) compared to the commercial varieties, indicating a pronounced dominance effect from PI161375 in shaping ovary morphology.

### 3.4. Detection of Pollen Tubes in the Style and Arrival in the Ovule In Vivo

A significant interaction was observed between melon accessions and hours after pollination (HAP) (*p* < 0.05). Pollen germination was evaluated at intervals of 1, 2, and 3 h after pollination. At 1 HAP, the commercial cultivar Piel de Sapo exhibited the highest pollen germination rate (Table 3), whereas commercial varieties Galia and Ouro, along with their hybrids and the exotic accession, showed the lowest germination percentages. At 2 HAP, all accessions except Piel de Sapo presented increased pollen germination, indicated by the presence of actively growing pollen tubes in vivo. By 3 HAP, pollen tube growth reached its maximum for all accessions, surpassing levels observed at 1 HAP. Notably, the exotic accession PI161375 demonstrated the greatest pollen tube development, with an average germination rate of 64.5%.

**Figure 4:**
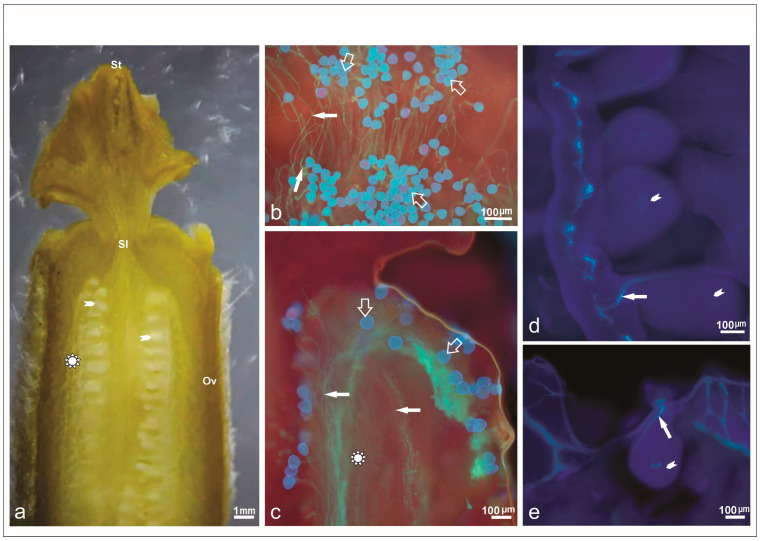
Hermaphrodite pistillate flower of *Cucumis melo*. (**a**) Longitudinal section of the gynoecium differentiated into stigma (St), style (Sl), and ovary (Ov) after hand pollination, with detail of the medium region (complex star) of the ovary (Ov) and ovule (arrowhead). (**b**) Pollen grains deposition (empty white arrow) on the stigma surface after staining for callose with aniline blue. (**c**) Pollen grains and growing pollen tubes (filled white arrow) exhibiting marked fluorescence three hours after hand pollination. Note the distance traveled beyond the medium region, which indicates pollen–pistil compatibility. (**d**) Pollen tube reaching the ovule after 3 h of pollination. See the path covered to the ovule. (**e**) Pollen tube arriving at the ovule.

The analysis of pollen tube growth and its trajectory along the style revealed a slower initial growth rate for the Galia, PI161375, Ouro, and hybrid PI161375 x Ouro accessions compared to other genotypes, a pattern maintained through 3 HAP (Table 3). Monitoring pollen behavior at the stigma clearly demonstrated that pollen grains germinated effectively and that pollen tubes successfully reached the ovules. A substantial increase in pollen tube number at the stigma was evident for all six evaluated genotypes (Figure 4). Among these, the genotypes Galia, Ouro, PI161375, and the hybrid PI161375 x Galia stood out, exhibiting the highest numbers of pollen tubes reaching the ovules. In contrast, the Piel de Sapo genotype showed no significant changes in pollen germination at the stigma throughout the evaluation period after pollination.

The evaluated genotypes showed an increase in the number of pollen grains reaching the ovule by three hours after pollination (Figure 4a), indicating that pollination duration is a crucial factor when assessing pollen grain germination on the stigma (Table 3). After three hours, pollen tubes had penetrated the stylar tissue, with some successfully arriving at the ovules.

Observations revealed that pollen tube growth at 1 HAP on the stigma (Figure 4b) was slower compared to the growth observed at 2 HAP (Figure 4c). By 3 HAP, pollen tubes had successfully entered the ovarian cavity (Figure 4d). At this stage, pollen tubes displayed a highly fluorescent, elongated callose plug at their apical ends within the ovules (Figure 4e). Notably, two distinct periods of callose deposition were observed during in vivo pollen tube growth following pollination (Figure 4e).

### 3.5. Internal Structures of Bisexual Flower

**Figure 5:**
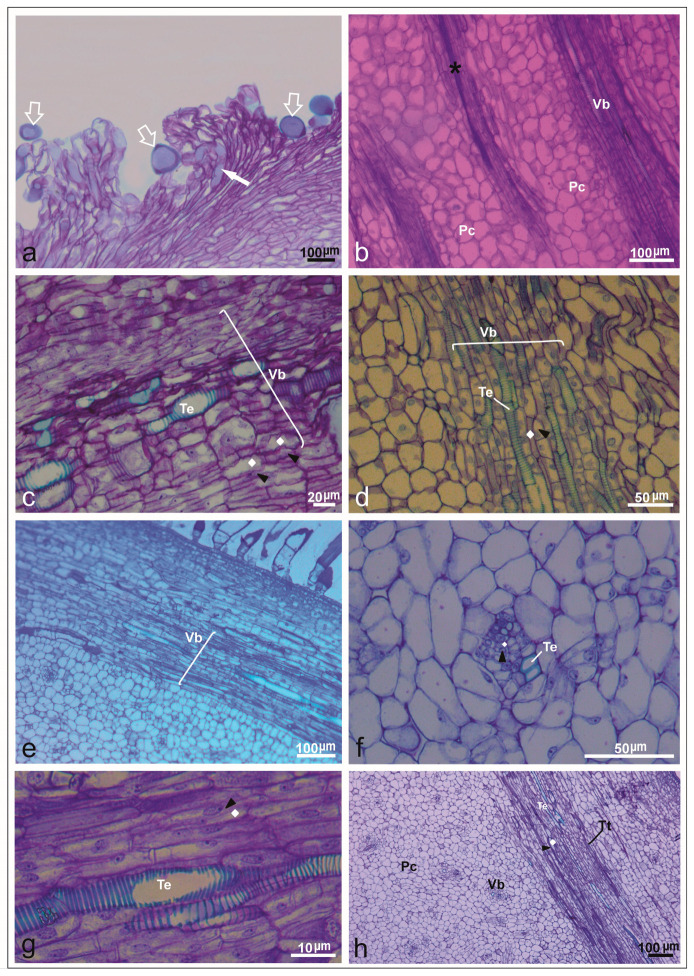
Ovary wall in longitudinal and transversal sections after pollination in *Cucumis melo* under a light microscope. (**a**) Longitudinal section of the upper region of a mature stigma with pollen grains (empty white arrow) and pollen tube germination (filled white arrow) in PI161375 x Galia accession. (**b**) Longitudinal section of the receptacle covered with parenchymatic cells (Pc), vascular bundle (Vb), and transmistting tissue (asterisk) in Ouro accession. (**c**) Vascular bundle (Vb): sieve tube (polyhedron) and companion cells (black arrowheads) with many vesicles accumulated together in PS accession. (**d**) Pistil flowers from Galia accession detailing vascular bundle (Vb): companion cells (black arrowheads), sieve tube (polyhedron), and parenchymal cells (Pc). (**e**) Longitudinal section of the upper region of a mature stigma with details of vascular bundle (Vb) and parenchymal cells from PI161375 accession. (**f**) Cross-section of ovary in PI161375 accession, showing collateral vascular bundle with sieve tube (polyhedron), companion cells (black arrowheads), tracheal elements (Te) of xylem, and parenchymal tissue. (**g**) Longitudinal section in Piel de Sapo accession, showing tracheal elements, companion cells (black arrowheads), sieve tube (polyhedron), and parenchymal cells (PC). (**h**) Longitudinal section of ovary showing transmitting tissue (asterisk), vascular bundles (Vb), and tracheal elements (Te) in PI161375 x Ouro hybrid.

After different intervals of post-pollination, abundant pollen tubes were present on the stigma, clearly converging within the style across all flowers examined (Figure 5a). These findings confirm a pronounced increase in pollen tube number reaching ovules, a trend consistent across all accessions studied. The evaluation revealed pollen germination within the internal style tissues, with pollen tubes extending actively through the transmitting tissue.

Histological examination identified prominent phloem tissues characterized by sieve tube elements and companion cells, along with distinct tracheary elements within ovarian tissues (Figure 5b,c). Internal epidermal layers containing vascular bundles, parenchyma, and well-developed transmitting tissues were particularly evident in PI161375 and Ouro accessions (Figure 5d,e). Mature pistils displayed extensive phloem, parenchyma, xylem cells, and transmitting tissues, underscoring that sugars and water are essential resources supporting pollen tube elongation (Figure 5f). Additionally, parenchymal cells, vascular tissues, and transmitting tissues were consistently observed within the stigmatic apex across all accessions (Figure 5g,h). Moreover, the principal phloem cell types identified in the commercial varieties and the exotic accession are detailed in Table 4.

## 4. Discussion

Floral characteristics and the variability of their descriptors are essential aspects of plant reproductive biology, playing a fundamental role in understanding genetic diversity among melon accessions. Knowledge of these floral traits is particularly valuable for effective germplasm management and facilitates informed decisions in plant breeding and genetic improvement programs. The present study was designed to elucidate the biological mechanisms underlying pollen grain germination in vivo, pollen tube growth through the style, and to examine how the timing following hand pollination influences pollen tube development across various melon accessions. Considering that hand pollination is a widespread practice in protected melon cultivation systems, assessing its efficiency becomes critical, as success can differ significantly depending on cultivar-specific responses.

### 4.1. Genetic Diversity in Floral Characteristics and Pollen Grain Viability

The Ward–MLM multivariate strategy proved effective in assessing genetic diversity and variability, integrating both quantitative and qualitative descriptors. This analytical approach is particularly valuable in evaluating floral, reproductive, and plant resistance traits, which are essential in plant breeding programs, as previously demonstrated in Brazilian melon accessions [25].

In the current study, 21 melon accessions were grouped into six clusters based on genetic distances. Notably, the Ouro accession grouped closely with the PI161375×Ouro intervarietal hybrid, a clustering driven primarily by their shared trait of low pollen tube growth in vivo. This grouping highlights the reliability of the multivariate analysis, given that the hybrid genotype inherently shares at least half of its allelic composition with the Ouro parental line. Likewise, the short genetic distance observed between groups 2 and 6 can be attributed to the common parentage involving genotype PI161375 in the hybrid assigned to group 6. Such consistency in the clustering of hybrids according to parental genotypes has been similarly reported in other species, including *Cucumis melo* and *Passiflora* spp. [37,38].

Regarding flower type, the evaluated melon germplasm consisted predominantly of andromonoecious plants (81%), with a smaller proportion (19%) classified as trimonoecious [7,39]. Although melon plants commonly exhibit andromonoecious or monoecious flowering patterns, four Brazilian accessions (A19, A35, A50, D2) presented the uncommon trimonoecious condition. According to [40], in andromonoecy, apart from losing the female part to form staminate flowers, the male part is retained in hermaphrodite flowers probably to offer pollen as a reward to pollinators, which, in turn, may help to retain pollinators on these flowers for a longer time, resulting in better deposition of pollen collected from staminate flowers and enhanced reproductive success.

This floral trait, considered mutant in cucurbits, has been previously observed in watermelon and zucchini lines subjected to high-temperature stress [41]. Its occurrence is linked to the androecy gene, which regulates female flower development by encoding a key enzyme (ACS11) involved in ethylene biosynthesis [42,43]. The expression of this genetic trait in the four Brazilian accessions underscores its relevance as a distinctive morphological characteristic within melon germplasm.

The melon phenology obtained in this study was similar to that reported by [10], which showed flowering started around 40 days after sowing, with 50% flowering observed between 55 and 60 days after planting. At the same time, flowering in melon is influenced by various environmental factors such as day length, temperature, stress, as well as endogenous genetic components, particularly ethylene biosynthesis pathways involved in bisexual flower formation and development. Understanding these factors provides opportunities for developing melon genotypes with earlier fruit production [44,45]. In plant breeding, understanding how reproductive success can be maximized, especially in fruiting species such as melon, is critical for productivity and genetic improvement.

In *Cucumis melo*, plants bearing different flower types (pistillate, staminate, and bisexual) typically grow concurrently. Flowering usually begins approximately 40 days after planting and extends for another 40 days, limiting the time available for controlled crossing in breeding programs [40]. In the present study, most evaluated accessions exhibited staminate flowering occurring within 30 to 34 days, while the appearance of hermaphrodite flowers occurred later, between 41 and 45 days. This overlap in sexual flower emergence likely enhances reproductive efficiency, as pollen grains produced by staminate flowers remain available during the emergence of hermaphrodite flowers, thus favoring successful pollination.

Female flowers remain receptive for approximately one day, necessitating that hand pollination be performed promptly on the day of flower opening. In *Cucumis sativus* L., pollen germination on hermaphrodite flowers has been reported to occur effectively across different flower maturity stages [46], underscoring the importance of precise timing in pollination strategies for melon cultivation.

For the commercial varieties Galia and Ouro, flowers exhibited smaller dimensions yet demonstrated higher pollen viability. Similarly, hybrids PI161375 × Galia and PI161375 x Ouro showed elevated pollen viability, although PI161375 × Ouro had a flower width smaller than the overall average. These findings indicate that the hybrids possess male fertility comparable to their parental genotypes, confirming that larger floral structures do not necessarily correlate with higher pollen viability [47]. It is well established that pollen grain germination and subsequent pollen tube growth are primarily influenced by the nutritional content and reserves stored within the pollen grain itself [48].

Within the cucurbit family, a substantial proportion of species exhibit dioecious reproductive systems. Nevertheless, *C. melo* displays distinctive floral biology, characterized by the presence of male, female, and hermaphrodite flowers. This particularity can give rise to notable phenotypic variation in fruits [49]. Moreover, external factors, especially ethylene exposure, can temporarily influence flower type expression in individual plants. The absence of stamens in female flowers, for instance, can lead to the production of larger and more elongated fruits [7].

Additionally, classifying melon germplasm according to flowering timing (early, medium, and late) provides valuable information for planning hybridization experiments and facilitates scientifically guided crossing strategies. Northeastern Brazil, the region with the highest melon production in the country, achieves multiple harvests annually [50], underscoring the practical importance of characterizing flowering time in Brazilian melon accessions with significant commercial potential.

### 4.2. Stigma’s Receptivity and Morphological Description

The receptivity obtained in this study was similar to that reported by [51,52], who investigated stigmatic receptivity in *Passiflora edulis* with the peroxidase enzyme method and obtained positive results. Also, ref. [53] evaluated stigma receptivity with the methodology of hydrogen peroxide in flowers of *Carica papaya* L. and found that stigma receptivity started before anthesis. According to [51], stigma receptivity is the ability to receive pollen, allowing it to adhere, hydrate, and germinate, which can be determined by evaluating the presence of peroxidase enzymes.

The six melon genotype groups exhibited variability in ovary morphology and stigmatic descriptors, with the exception of stigma length. The selection of these accessions was intentional, as they belong to distinct botanical groups, allowing the exploration of pollination responses between commercial varieties and exotic accessions. Importantly, all Brazilian accessions, hybrids, commercial varieties, and the exotic accession consistently demonstrated high pollen viability coupled with effective stigma receptivity. In the Cucurbitaceae family, the interval between pollen grain adhesion to the stigma and pollen tube emergence is typically very short, ranging from seconds to minutes, and pollen viability decreases rapidly due to rapid water loss once separated from the anther [50].

The commercial varieties Galia and Ouro presented higher mean values for floral traits, particularly staminate and pistillate flower descriptors, compared to their hybrids. Previous studies suggest that the inheritance patterns observed in F1 hybrids frequently involve non-allelic interactions, with certain traits predominantly governed by additive or dominance genetic effects. Furthermore, traits such as fruit number per plant, fruit weight per plant, and average fruit weight typically exhibit high heritability values, supporting the reliability of selection in breeding programs [54].

Correlation analyses have established strong relationships between ovary morphology and subsequent fruit shape. A significant positive correlation between ovary shape and ovary length indicates that ovary shape traits are predominantly determined by length-related genes [55]. Similarly, ref. [56] reported robust correlations between ovary and fruit shape in 14 genetically distinct melon lines, suggesting that genes active early in ovary development broadly influence fruit morphology in *C. melo*. Additionally, the exotic accession PI161375 harbors alleles beneficial for earliness, fruit weight, and sugar content, highlighting its potential as a valuable genetic resource for melon improvement [57].

### 4.3. Detection of Pollen Tubes in the Style and Arrival in Ovules In Vivo

Using epifluorescence microscopy in hybrids, commercial varieties, and the exotic accession PI161375, we found a continuous increase in the number of pollen tubes growing along the style at three distinct time points after pollination. This progressive growth reflects the dynamic nature of pollen tube elongation, a highly regulated cellular process dependent on the organization of the actin cytoskeleton and the availability of external calcium reserves within the pistil tissues [41,58,59].

Our work is similar to that of [60], who demonstrated that the pollen tubes do indeed travel through the transmitting tissue. In their study, serial sections of the ovary of *C. intybus* were treated with decolorized aniline blue and then observed under a fluorescence microscope, where the callose of the pollen tubes glows within the transmitting tissue. The same author found that callose is stained by the fluorochrome aniline blue. Therefore, pollen tubes are often visualized by aniline blue staining as tube-like structures interdigitated with callose plugs.

Comparable observations were reported by [61], who documented slow but steady pollen tube growth in *C. melo* PI 124111F, evident as early as 30 min and particularly pronounced 5.5 h after cross-pollination. These findings reinforce the importance of temporal monitoring to fully capture the dynamics of pollen tube development and fertilization potential in melon accessions.

The variation in pollen tube numbers observed among genotypes may result from physical or physiological constraints, such as the progressive depletion of reserves and limited space available for pollen tube growth within the style’s transmitting tissue [62]. Additionally, genetic interactions between the pollen tube gametophytes and the surrounding sporophytic tissues of staminate flowers may also contribute to these differences [13,63,64].

In melon, once pollen from the same or a different flower is deposited on the stigma, germination typically occurs within 30 min under favorable environmental conditions. Pollen grains germinate and elongate through a tip growth mechanism, achieving elongation rates of up to 1.0 cm per hour, which make pollen tubes among the fastest-growing plant cells [65]. Similarly, ref. [66] reported that in *Cucurbita pepo*, the number of pollen tubes increased over time but gradually decreased as the distance traveled through the style increased, likely due to similar physiological limitations.

The progressive increase in pollen tube numbers within the style and their successful arrival at the ovules by 3 h after pollination (HAP) provides clear evidence of gametophytic compatibility among the six genotypes evaluated. This result highlights the efficiency of pollen–pistil interactions and supports the reproductive potential of the studied accessions.

Similar dynamics have been described in other species, where callose deposition serves as an indicator of active reproductive processes. Ref. [67] observed intense callose fluorescence during the globular embryo stage, coinciding with the initiation of basic tissue formation. As embryo development progresses, callose is rapidly degraded, reflecting its temporary role in early developmental stages. Consistently, callose accumulation is commonly detected in developing megaspores of flowering plants and plays critical roles in diverse biological processes, including the formation of specialized cell walls or cell wall-associated structures during specific phases of growth and differentiation [68,69].

In tomato (*Solanum lycopersicum*), ref. [70] reported that pollen tubes emitting green-blue fluorescence from aniline blue staining of callose successfully entered the ovary and reached the ovules at 24 and 48 h after pollination, further illustrating the conserved role of callose in monitoring pollen tube progression and fertilization across species.

### 4.4. Internal Hermaphrodite Structure

This study provided detailed insights into the histological structure of the pistil, focusing on how pollen grains adhere to the stigma surface, germinate, and subsequently emit pollen tubes that grow in vivo through the transmitting tissue along the pistil. The presence of papillae on the stigma surface was evident, particularly in the exotic accession PI161375, suggesting specialized adaptations that may enhance pollen adhesion and germination efficiency. Our work agrees with [60], which reported that pollen tube growth in the style is heterotrophic and that this growth is at the expense of the reserves of the transmitting tissue, suggesting that the availability of these reserves may affect the number of pollen tubes growing within the style. According to [63], the number of pollen tubes found in a style may be limited by physical constraints. Also, ref. [60] repeatedly observed a progressive reduction in the width of the transmitting tissue from the stigma.

Histological analyses revealed that the pistil is predominantly composed of phloem and parenchymal cells, consistent with previous findings reported by [16]. Observations confirmed that, in melon, successful pollen tube penetration into the ovary requires a minimum of three hours after pollination for the tubes to traverse the style, and that the transmitting tissue functions as the primary site of competition among pollen tubes, underscoring its crucial role in fertilization dynamics [58,62].

Moreover, both the duration of pollen tube growth and the quantity of pollen grains deposited on the stigma play fundamental roles in reproductive success. Ideally, the number of viable pollen grains should exceed the number of ovules in the ovary to maximize fertilization potential [62].

The anatomical observations of melon flowers after manual pollination allowed us to confirm that the pistil is composed of phloem and xylem vessels, structures essential for transporting nutrients that sustain pollen tube growth. This vascular system ensures the continuous supply of resources necessary for the metabolic demands of the elongating pollen tubes.

In cucumber, ref. [71] characterized a specific hexose transporter, CsHT1, identified as a hexose/H^+^ symporter with high affinity for glucose. Their study demonstrated that overexpression of CsHT1 in pollen promotes pollen tube growth in media containing glucose or galactose, while its downregulation significantly reduces growth under the same conditions. Similarly, ref. [15] reported that CsHT1 is in the plasma membrane and is exclusively transcribed during pollen development. Its expression is evident at both transcriptional and translational levels during pollen grain germination and pollen tube growth, highlighting the critical role of sugar transport in supporting successful fertilization processes.

However, even when pollen is successfully delivered to a receptive stigma, fertilization is not guaranteed, as pollen viability and the capacity to reach and fertilize the ovule may still be compromised by physiological or environmental factors [71].

## 5. Conclusions

Brazilian accessions, hybrids, commercial varieties, and the exotic accession of melon were distributed into six genetically distinct groups based on variability in floral characteristics and reproductive traits. All genotypes exhibited high pollen germination capacity, although pollen tube growth in vivo varied among genotypes depending on the time elapsed after hand pollination.

Anatomical analyses revealed the presence of well-developed transmitting tissues and vascular bundles along the pistil in all six genotypes. These structural features likely contributed to the higher success rate of pollen tube progression and ovule fertilization observed 3 h after pollination, particularly in the exotic accession.

Further studies on the reproductive biology of melon, especially those focusing on Brazilian germplasm, are essential to support future breeding programs and the strategic use of genetic resources aimed at improving reproductive efficiency and fruit production.

## Figures and Tables

**Table 1:** Distribution of quantitative values for staminate flower descriptors and reproductive pollen viability in different melon genotypes, and qualitative flowering time and flower reproductive type characteristics analyzed using the Ward–MLM multivariate strategy.

Ward–MLM Group	Accessions/Genotypes	PL	FW	RL	BL	RW	FL	PGV	MFT	BFT	FRT
I	A9	15.84 ± 0.68 a	31.16 ± 0.28 a	5.75 ± 0.22 c	3.73 ± 0.52 b	3.07 ± 0.11 b	20.42 ± 0.50 b	95.75 ± 1.11 a	PRE	PRE	SB
	A30	15.21 ± 0.06 a	31.78 ± 0.9 a	5.64 ± 0.11 c	3.23 ± 0.02 c	3.05 ± 0.09 b	20.45 ± 0.53 c	97.00 ± 0.14 a	PRE	PRE	SB
	A35	14.40 ± 0.70 b	29.70 ± 1.18 b	5.20 ± 0.33d	2.67 ± 0.54 c	2.75 ± 0.21 b	18.88 ± 1.04 c	97.83 ± 0.97 a	PRE	PRE	SPB
	I Group mean	15.15	30.88	5.53	3.21	2.96	19.92	96.86	-	-	-
II	A6	14.26 ± 0.52 b	28.59 ± 0.07 b	6.63 ± 0.34 b	4.20 ± 0.56 b	3.50 ± 0.15 a	21.14 ± 0.05 b	98.33 ± 0.14 a	MED	PRE	SB
	A18	14.18 ± 0.60 b	28.36 ± 0.16 b	6.20 ± 0.09 b	4.13 ± 0.19 b	3.21 ± 0.14 b	20.53 ± 0.56 c	98.50 ± 0.03 a	MED	PRE	SB
	A44	15.04 ± 0.26 b	28.06 ± 0.46 b	6.63 ± 0.12 b	3.47 ± 0.47	3.79 ± 0.44 a	21.71 ± 0.62 b	98.41 ± 0.06 a	MED	MED	SB
	PI161375	15.65 ± 0.87 a	29.05 ± 0.53 b	5.71 ± 0.34 c	3.95 ± 0.01 b	2.90 ± 0.45 b	20.99 ± 0.01 b	98.66 ± 0.19 a	MED	MED	SB
	II Group mean	14.78	28.52	6.29	3.94	3.35	21.09	98.47	-	-	-
III	A1	16.50 ± 0.45 a	34.99 ± 0.85 a	5.55 ± 0.77 c	5.19 ± 0.10 a	3.50 ± 0.08 a	21.77 ± 1.28 b	97.50 ± 1.22 a	MED	MED	SB
	A7	17.29 ± 0.34 a	35.01 ± 0.87 a	7.54 ± 1.22 a	5.36 ± 0.27 a	3.55 ± 0.13 a	25.11 ± 2.06 a	98.66 ± 0.06 a	MED	MED	SB
	A24	15.94 ± 1.01 a	32.61 ± 1.53 a	6.05 ± 0.27 b	5.02 ± 0.07 a	3.41 ± 0.01 a	21.19 ± 1.86 b	99.66 ± 0.94 a	MED	MED	SB
	A27	18.07 ± 1.12 a	33.93 ± 0.21 a	6.12 ± 0.20 b	4.78 ± 0.31 a	3.22 ± 0.20 b	24.11 ± 1.06 a	99.08 ± 0.36 a	MED	MED	SB
	III Group mean	16.95	34.14	6.32	5.09	3.42	23.05	98.72	-	-	-
IV	A19	13.38 ± 3.57 b	27.88 ± 0.75 b	5.56 ± 0.19 c	3.25 ± 0.13 c	2.96 ± 0.21 b	18.15 ± 1.01 c	98.00 ± 0.44 a	LAT	PRE	SPB
	A50	13.93 ± 3.02 b	27.65 ± 0.52 b	5.46 ± 0.29 c	2.72 ± 0.40 c	3.10 ± 0.07 b	19.57 ± 0.41 c	97.00 ± 0.56 a	LAT	MED	SPB
	Ouro	12.86 ± 4.09	25.41 ± 1.72 c	5.78 ± 0.03	2.69 ± 0.43 c	3.24 ± 0.07 b	18.88 ± 0.28 c	99.00 ± 1.44 a	LAT	MED	SB
	PI161375 x Ouro	14.66 ± 2.29 b	27.58 ± 0.45 b	6.19 ± 0.44 b	3.82 ± 0.70 b	3.39 ± 0.22 a	20.03 ± 0.87 c	96.25 ± 1.31 a	LAT	MED	SB
	IV Group mean	16.95	27.13	5.75	3.12	3.17	19.16	97.56	-	-	-
V	Piel de sapo (PS)	13.01 ± 0.60 b	23.46 ± 0.16 c	5.71 ± 0.12 c	5.21 ± 1.01 a	3.68 ± 0.16 a	19.66 ± 0.62 c	96.25 ± 0.87 a	LAT	LAT	SB
	Galia	11.81 ± 0.60 b	23.78 ± 0.16 c	5.47 ± 0.12 c	3.19 ± 1.01 c	3.35 ± 0.17 a	18.41 ± 0.63 c	98.00 ± 0.88 a	LAT	LAT	SB
	VI Group mean	12.41	23.62	5.59	4.20	3.52	19.04	97.12	-	-	-
VI	A11	15.66 ± 0.59 a	31.12 ± 1.32 a	5.16 ± 0.21d	4.00 ± 0.14 b	3.34 ± 0.12 c	20.45 ± 0.20 c	97.25 ± 0.72 a	MED	PRE	SB
	A43	14.73 ± 0.34 b	28.38 ± 1.42 b	4.61 ± 0.79d	4.12 ± 0.26 b	2.93 ± 0.29 b	17.30 ± 2.95 c	97.00 ± 0.97 a	MED	LAT	SB
	D2	14.87 ± 0.20 b	29.67 ± 0.13 b	5.76 ± 0.36 c	3.31 ± 0.55	3.22 ± 0.00 b	21.20 ± 0.95 b	98.91 ± 0.94 a	PRE	LAT	SPB
	PI161375 x Galia	15.02 ± 0.05 b	30.04 ± 0.24 b	6.08 ± 0.68 b	4.02 ± 0.16 b	3.39 ± 0.17 a	22.00 ± 1.75 b	98.75 ± 0.78 a	LAT	LAT	SB
	VI Group mean	15.07	29.80	5.40	3.86	3.22	20.25	97.97	-	-	-
	Total group mean	14.87	29.44	5.86	3.91	3.26	20.57	97.78	-	-	-
	CV%	12.8	12.77	8.19	25.43	8.84	11.48	1.82	-	-	-

Different lowercase letters denote significant differences at *p* < 0.05 by the Scott–Knott test are as follows: PL, petal length; FW, flower width; RL, receptacle length; BL, bract length; RW, receptacle width; FL, flower length; PGV, pollen grain viability; CV%, coefficient of variation. MFT, male flowering time after sowing: PRE, precocious (<29 days after sowing); MED, medium (30 to 34 days after sowing); and LAT, late (>35 days after sowing). BFT, bisexual flowering time after sowing: PRE, precocious (<40 days after sowing); MED, medium (41 to 45 days after sowing); and LAT, late (>46 days after sowing). FRB, flower reproductive types: B, bisexual; P, pistillate; and S, staminate. Genotypes identified with the letter “A” followed by numbers are accessions of the germplasm bank; Galia and Ouro are commercial cultivars; PI161375 x Galia and PI161375 x Ouro are hybrids.

**Table 2:** Mean values for pistillate flower descriptors in six different melon genotypes.

Accessions/ Genotypes	Descriptors			
	Ovary Length	Ovary Width	Stigma Length	Stigma Width
PI161375	16.68 ± 1.31 c	8.59 ± 0.35 c	5.13 ± 0.40 a	5.12 ± 0.83 a
Piel de Sapo	21.07 ± 3.07 b	11.05 ± 2.12 a	4.82 ± 0.09 a	3.93 ± 0.36 b
Ouro	15.13 ± 1.75 d	7.15 ± 1.77 d	4.68 ± 0.05	4.60 ± 0.31 a
PI161375 x Ouro	17.24 ± 0.74 c	9.53 ± 0.60 b	4.37 ± 0.36 a	4.14 ± 0.15 b
Galia	13.18 ± 4.80 d	8.44 ± 0.49 c	4.60 ± 0.13 a	3.51 ± 0.78 b
PI161375 x Galia	24.65 ± 6.66 a	8.81 ± 0.12 c	4.78 ± 0.05 a	4.46 ± 0.17 b
CV%	8.39	7.66	7.87	10.55
Mean	17.99	8.93	4.73	4.29

Different lowercase letters denote significant differences at *p* < 0.05 by the Scott–Knott test. CV%, coefficient of variation.

**Table 3:** Means of interaction between genotypes and different times after pollination: pollen grains germinated and pollen tubes arriving in ovules.

Accessions/ Genotypes	Pollen Tube Growth In Vivo			Pollen Tube Arriving in Ovule		
	1 HAP	2 HAP	3 HAP	1 HAP	2 HAP	3 HAP
PI161375	19.75 ± 0.03 c	56.75 ± 25.58 a	64.50 ± 26.92 a	3.50 ± 1,79 b	4.00 ± 0.25 b	7.50 ± 1.05 a
Piel de Sapo	38.25 ± 18.92 b	39.75 ± 8.58 b	49.00 ± 11.42 b	1.50 ± 0.21 c	2.00 ± 1.75 c	3.67 ± 2.78 b
Ouro	9.50 ± 10.05 c	25.75 ± 5.42 b	25.75 ± 11.83 c	1.25 ± 0.46 c	2.75 ± 1.00 b	6.75 ± 0.03 a
PI161375 x Ouro	9.00 ± 10.54 c	11.50 ± 16.67 c	23.75 ± 11.83 b	0.00 ± 1.71 c	0.00 ± 3.75 c	4.25 ± 2.20 b
Galia	14.50 ± 5.07 c	20.75 ± 10.42 c	28.00 ± 9.58 c	3.50 ± 1.79 b	6.00 ± 2.25 a	8.50 ± 2.05 a
PI161375 x Galia	26.50 ± 6.92 b	32.50 ± 1.33 b	34.50 ± 3.08 b	0.50 ± 1.21 c	7.75 ± 4.00 a	8.00 ± 1.55 a

Different lowercase letters columns denote significant differences at *p* < 0.05 by the Scott–Knott test. HAP (hour after pollination).

**Table 4:** Phloem cell types and their functions in different *Cucumis melo* accessions.

Phloem Cell Type	Functions	Accessions
Sieve tube elements	Translocation of sugar, amino acids and hormones	PI161375; Piel de sapo
Companion cells	Metabolic support, phloem loading/unloading	Galia; Piel de sapo
Parenchyma	Storage and synthesis	PI161375; Piel de sapo; Ouro

## Data Availability

The original contributions presented in this study are included in the article. Further inquiries can be directed to the corresponding author.
